# Editorial

**Published:** 1872-05-15

**Authors:** 


### Medical Examiner.

*A Semi-Monthly Journal of Medical Sciences.*

EDITED BY

N. S. DAVIS, M. D., AND F. H. DAVIS, M. D.

Chicago, May 15th, 1872.

### EDITORIAL.

Illinois State Medical Society.—The annual meeting of this Society was held in Rock Island, commencing at 10 o'clock a.m. of Tuesday, May 21st, and continued until 10 o'clock p.m. of Wednesday.

Although the number in attendance was not as large as at Peoria the year previous, yet it was above the average attendance at our annual meetings.

The days were pleasant, the accommodations good, and the members devoted themselves assiduously to the transaction of business, holding three sessions the first day and two the second. A large proportion of the Standing and Special Committees, appointed the year previous, failed to make any report; yet the number of reports and papers was quite equal to that of previous meetings, and each elicited more or less intelligent and interesting discussion. We shall give an abstract of the more interesting part of the proceedings in our next number. On the afternoon of the second day, the members enjoyed an excursion to Moline, thence to the island, where the ladies of Rock Island had provided a most elegant and bountiful *pic-nic* entertainment, after doing full justice to which, the company crossed the great river, and were very cordially received by the profession of Davenport, and escorted through that flourishing city to the bluffs, from which they enjoyed one of the most beautiful and extensive landscape views that could be presented to the eye. At 8 o'clock p.m., the hall was well filled with members of the profession and citizens, to hear a public address from Dr. A. L. McArthur, of Rockford. The address was well written, and delivered in good style.

The aim of the speaker was to show the close relation between physical health or perfect physical development and the mental and moral actions of men; and as an inference, that all great improvements in the elevation of the race must be founded on a better understanding and more general observance of the physiological laws governing physical development and health. After the lecture, the Society proceeded with its ordinary business, but as many of the members wished to leave for home on the R. R. train at 10:30 p.m., the closing work was done somewhat hastily, and some items of importance were wholly omitted. We were glad to see delegates present from the State Medical Societies of Iowa and Wisconsin.

Minutes of the Amer. Med. Association.—We are informed that the official record of proceedings of the recent meeting of the American Medical Association, is now in press, and will soon be ready for distribution. It will be in pamphlet form, price fifty cents per copy. All who desire one or more copies, should enclose the amount to the permanent Secretary, Dr. Wm. B. Atkinson, 1400 Pine street, Philadelphia.

Cholera.—We see announcements that one or more cases of cholera have occurred in Paris, France, early this spring, and that the disease appeared in considerable severity at Ramionka, in Russia. We should not be disappointed if the cycle of atmospheric conditions favorable for its development, should reach this country during the coming summer.

Chicago College of Pharmacy.—At the meeting held March 6th, the retiring President, Mr. E. H. Sargent, in his annual address, spoke feelingly of the sympathy extended by the pharmacists of this country and Europe to this College, after the disastrous conflagration. Resolutions of thanks to all contributors were adopted.

The following officers were elected: George Buck, President; Th. H. Patterson, J. W. Mill, Vice-Presidents; G. M. Hambright, Secretary; A. C. Vanderburgh, Treasurer; A,

E. Ebert, Corresponding Secretary; W. F. Blocki, Henry Biroth, N. Gray Bartlett, E. H. Sargent, J. C. Borcherdt, J. M. Hirsh, J. H. Mead, Thos. Whitfield, Jul. H. Wilson, Thos. N. Jamieson, Trustees.

INDIANA STATE MEDICAL SOCIETY.—Through the kindness of the Secretary, Dr. S. A. Woolen, of Indianapolis, we have received papers containing the record of proceedings of the recent meeting of the State Medical Society of Indiana, the most interesting portions of which we will give to our readers in the next number of THE EXAMINER.

